# Screening, Diagnosis, and Management of Patients With Alcohol Use Disorders at Bwindi Community Hospital, Uganda

**DOI:** 10.3389/fpubh.2018.00148

**Published:** 2018-05-24

**Authors:** Yusufu Kuule, Andrew E. Dobson, Anthony D. Harries, Birungi Mutahunga, Alex G. Stewart, Ewan Wilkinson

**Affiliations:** ^1^Church of Uganda, Bwindi Community Hospital, Kanungu, Uganda; ^2^Centre for Operational Research, International Union Against Tuberculosis and Lung Disease, Paris, France; ^3^London School of Hygiene and Tropical Medicine, London, United Kingdom; ^4^College of Life and Environmental Science, University of Exeter, Exeter, United Kingdom; ^5^The Institute of Medicine, University of Chester, Chester, United Kingdom

**Keywords:** alcohol screening, alcoholics anonymous, detoxification, mental health, operational research, SORT IT

## Abstract

**Introduction:** The harmful use of alcohol is a growing global public health concern, with Sub-Saharan Africa at particular risk. A large proportion of adults in Uganda consume alcohol and the country has a high prevalence of alcohol use disorders (AUD), almost double that for the African region as a whole. Bwindi Community Hospital, in rural western Uganda, recently introduced a program of screening, diagnosis and management of AUD and we assessed how this worked.

**Methods:** This was a cross-sectional study in three departments (out-patients, adult in-patients and sexual & reproductive health) of Bwindi Community Hospital assessing numbers of patients screened, diagnosed and treated with AUD between January 2014 and June 2017. Data sources included the hospital electronic data base and departmental case files. Frequencies and proportions are reported and odds ratios used to compare specific factors associated with medical interventions.

**Results:** Altogether, 82,819 patients attended or were admitted to hospital, of whom 8,627 (10.4%) were screened and 273 (3.2%) diagnosed with AUD. The adult in-patient department recorded the largest number with AUD (*n* = 206) as well as a consistent increase in numbers in the last 18 months of the study. Of those with AUD, there were 230 (84%) males, 130 (48%) aged 36–60 years, and 131 (48%) with medical non-alcohol related diagnostic categories. Medical/supportive interventions included guidance and counselling to 168 (62%), community social support to 90 (33%), mental health service referrals for 75 (27%), detoxification for 60 (22%) and referral to Alcoholics Anonymous for 41 (15%). There were 36 (15%) patients who received no medical/supportive interventions, with significantly higher proportions in patients with surgical alcohol-related disease and pregnancy-related conditions (*P* < 0.05).

**Conclusion:** Bwindi Community Hospital has implemented a program for AUD in three departments, with most individuals screened and managed in the adult in-patient department. While a variety of interventions were given to those with AUD, 15% received no intervention and this deficiency must be addressed. Program performance could improve through better screening processes, ensuring that 100% of those with AUD receive a medical/supportive intervention and raising public awareness.

## Introduction

Alcohol is the most commonly used recreational drug in the world, with nearly 40% of the world's population aged 15 years and older estimated to have drunk alcohol in the preceding 12 months (1). In 2012, about 6% of all global deaths (3.3 million) were attributable to alcohol consumption, affecting males more than females, and these are additional to alcohol-induced morbidity and injuries (1). The growing problem with alcohol consumption has led to its inclusion in the health-related Sustainable Development Goal (SDG 3.5) which aims to strengthen the prevention and treatment of substance abuse, including narcotic drug abuse and harmful use of alcohol (2). In turn, tackling alcohol will help the global community to reduce by one third the premature mortality that occurs from non-communicable diseases (SDG3.4) (2).

There are more than 200 ICD-10 (International Classification of Diseases, 10th Edition) disease and injury codes for which alcohol consumption is an important cause, and more than 30 include alcohol in their name or definition (1). Of these 30, alcohol use disorders (AUD) are the most significant and describe a group of physical and mental disorders which occur when alcohol is used inappropriately and over a long period of time (1). Untreated AUD all over the world can lead to frequent contacts with health care systems and can be associated with considerable human and societal costs (3–8). In 2010, the World Health Organization (WHO) launched a global strategy to reduce the harmful use of alcohol that included public awareness, community action, drink-drive policies, pricing policies, dealing with alcohol intoxication and monitoring and surveillance (9). To help take interventions down to the primary care level, WHO further developed a practical guide (AUDIT—Alcohol Use Disorders Identification Test) to identify persons with hazardous and harmful patterns of alcohol consumption, using a standardized scoring system, with the goal of helping risky drinkers reduce or cease alcohol consumption and thereby avoid the harmful consequences of their drinking (10).

## Background and rationale

Recent publications have highlighted the growing and harmful use of alcohol in sub-Saharan Africa (11, 12) and the fact that the alcohol beverage industry has identified the continent as a key area for alcohol market growth (13). This growing public health threat affects all countries in the region and especially Uganda. The most recent WHO Report of 2014 highlighted that AUD affected 5.8% of the Uganda population aged 15 years and above (10% in males and 1.5% in females) which was considerably higher than the average for the African region at 3.3% (1). A more recent study using data from Uganda's non-communicable diseases risk factor survey found that 27% of Ugandan adults were current alcohol users and that almost 10% had AUD (14). Nearly 70% of liver cirrhosis and 6% of road traffic accidents in the country are attributable to alcohol (1), and there is a growing problem of alcohol use and its risks in students and in people co-infected with HIV (15–17). Despite this looming threat, there is no current national policy or action plan to tackle the alcohol problem, although there is national government support for community action along with monitoring and surveillance (1). Legislation on alcohol purchase and consumption in Uganda has little impact on levels of consumption. The laws in the country were enacted in 1965, and any fine that is imposed is now of low value due to inflation and thus is no deterrent. There are also many types of locally made alcoholic drinks that are not covered under the enacted laws as they were not available when the legislation was passed. The minimum age for purchase or consumption of alcohol is 18 years, but younger people can easily obtain alcohol (18).

Bwindi Community Hospital (BCH) is a Private Not for Profit Hospital under the Kinkizi Diocese of the Church of Uganda in Kanungu District, South Western Uganda. Its immediate catchment area covers three sub counties which together have a population of about 70,000 living in 101 villages (19). BCH is one of few non-governmental organizations in Uganda that has been offering Mental Health Services, including the identification and management of AUD and alcohol rehabilitation services, since January 2014. The program is set up in such a way that every adult patient who comes to the hospital is supposed to be screened for an AUD using AUDIT (20). The AUDIT assessment is mainly carried out in out-patient, the adult in-patient and the sexual and reproductive health services departments (the latter serving maternal health, ante-natal and post-natal care and family planning). The assessment is done by nurses and midwives, clinical officers and medical officers in collaboration with the mental health team. Patients diagnosed with AUD are supposed to be given specific interventions.

AUD are frequently undetected and untreated in health care settings in Africa and elsewhere (6–8, 21), and as a result patients with AUD end up not receiving appropriate treatment for their condition. These system failures have a number of reasons that include lack of knowledge, little available time to spend with individual patients or poor health care worker attitudes. They may also be due to patients with AUD attending or being admitted to health units with other physical and mental disorders that take higher priority over their AUD. Since the program in BCH for screening, diagnosis and management of AUD started, there has been no systematic assessment about how the processes have worked or about the numbers diagnosed with AUD or the treatments being offered. The current study was therefore undertaken at BCH, Uganda, to determine the numbers and proportions of patients diagnosed with AUD and the numbers who received appropriate medical and supportive interventions. Specific objectives were to determine among persons aged 15 years and above attending the out-patient, the adult in-patient and the sexual & reproductive health departments between January 2014 and June 2017: (i) numbers and proportions of patients screened and diagnosed with AUD; (ii) demographic and clinical diagnostic categories of those diagnosed with AUD; and (iii) medical and supportive interventions given to those with AUD and in relation to baseline characteristics.

## Essential elements of the intervention

The procedures for screening and managing AUD are standardized and uniform across all three departments. Every person aged 15 years and above attending the out-patient department or admitted to the adult in-patient or sexual & reproductive health departments should be screened by a nurse, doctor, clinical officer or a member of the mental health team for AUD using the WHO-based Alcohol Use Disorder Identification Test (AUDIT) (10, 20). The AUDIT questionnaire has ten questions (each scoring from 0 to 4) related to frequency of drinking alcohol, number of units consumed, timing of first alcohol drink in the day, behavioral issues, injuries, forgetfulness and whether family and friends have been concerned. The total scoring places people in four possible categories: 0–7 = lower risk; 8–15 = increased risk; 16–19 = higher risk; and 20 and above = possible dependence. Anyone with a score of 8 or higher is regarded as having AUD (10).

A protocol for management of individuals with AUD was developed in the hospital, based on the WHO recommended approach (22). Anyone diagnosed with AUD should be offered medical and supportive interventions that include: brief guidance and counselling mainly given by nurses, midwives, doctors, clinical officers and members of the mental health team and social support from community and family members. Other interventions which may be offered include: detoxification that includes medication to avoid alcohol withdrawal symptoms (oral or parenteral vitamin B1 supplementation (thiamine), benzodiazepines, and antipsychotic medications if needed) (22); referral if deemed necessary to the mental health team; and referral to Alcoholics Anonymous (AA) Groups, eight of which have been established and function in the community and one of which comes to the hospital once a week to see referred clients. The offering of these interventions is based on decisions made by the clinical and nursing staff about each patient.

When the system was first set up in 2014, the details of every patient who attended the out-patient department and was screened and diagnosed with AUD were entered into a hospital-based electronic register by one of the attending health care workers. In the adult in-patient and sexual & reproductive departments, details were recorded in the patient case files. Since April 2017, the hospital has also introduced an electronic system for adult in-patients whereby a clinician cannot discharge a patient without undertaking a screening for AUD.

## Methods

### Study design

This is a retrospective cross-sectional study using routinely collected data.

### Setting

Uganda is a land-locked country in East Africa with an estimated population of 41 million people in 2016 (23, 24). The country is divided into 114 districts with 84% of the population living in rural areas and 20% living in poverty. Life expectancy was 60 years in 2016. Health coverage in the urban and rural areas is provided by both government and private entities. Primary health facilities play a more prominent role in the rural areas. The study site was Bwindi Community Hospital (BCH), which is a Private Not for Profit (PNFP) Hospital under Kinkizi Diocese of the Church of Uganda in Kanungu District in South Western Uganda. Its immediate catchment area covers the three sub counties of Kayonza, Kanyantorogo and Mpungu, which together have a population of about 70,000 living in 101 villages (18).

### Study population

All persons aged 15 years and above who were screened for and diagnosed with AUD at BCH, Uganda, between January 2014 and June 2017 were included in the study.

### Data variables and sources of data

Data variables included: hospital department (out-patient, adult in-patient, sexual and reproductive health); date; age, sex, screened for AUD; AUD score; diagnosed with AUD (score on AUDIT = 8 or more); clinical diagnostic category; medical and supportive intervention given; type of intervention (brief guidance and counselling, social support from community members, referral to mental health team, detoxification, referral to AA group). Sources of data included (a) the hospital electronic database for out-patients and for adult in-patients between April and June 2017, and (b) hospital case files for adult in-patients (before April 2017) and patients admitted to sexual & reproductive health. Data were collected into a structured proforma using EpiData software (version 4.0.1.44, EpiData Association, Odense, Denmark).

### Analysis and statistics

The data were analyzed in EpiData (version 2.2.2.186, EpiData Association, Odense, Denmark) using frequencies and proportions. Characteristics of patients with AUD who did not receive medical or supportive interventions were compared using the chi-square with results presented as odds ratios (OR) and 95% confidence intervals. Levels of significance were set at *P* < 0.05.

### Ethics issues

Ethics approval was obtained from the Health and Scientific Committee, Bwindi Community Hospital, Kanungu, Uganda and from the Ethics Advisory Group, International Union Against Tuberculosis and Lung Disease (The Union), Paris, France.

## Results

The number of persons aged 15 years and above attending one of the three departments at BCH along with numbers and proportions screened and diagnosed with AUD are shown in Table 1. Less than 5% of persons attending the out-patient department were screened and <2% of those screened were diagnosed with AUD. In contrast, over half of those admitted to the adult in-patient or sexual & reproductive health departments were screened. Nearly one in 10 adult in-patients was diagnosed with AUD but there were few women diagnosed in the sexual & reproductive health department. The largest numbers diagnosed with AUD were in the adult inpatient department.

**Table 1:** Screening and diagnosis of alcohol use disorders in three different departments of Bwindi Community Hospital, Uganda, between January 2014 and June 2017.

**Characteristics**	**Out-patient department**		**Adult in-patient department**		**Sexual and reproductive health department**		**Total**	
	***N***	**(%)**	***n***	**(%)**	***n***	**(%)**	***n***	**(%)**
Hospital attendances or admissions	72,723	(100)	4,285	(100)	5,811	(100)	82,819	(100)
Screened for alcohol use disorder	3,040	(4.2)^a^	2,312	(54.0)^a^	3,275	(56.4)^a^	8,627	(10.4)^a^
Diagnosed with alcohol use disorder	55	(1.8)^b^	206	(8.9)^b^	12	(<1)^b^	273	(3.2)^b^

a *Percentage of patient attendances or admissions who were screened for Alcohol Use Disorder*.

b *Percentage of patients screened who were diagnosed with Alcohol Use Disorder*.

**Figure 1:**
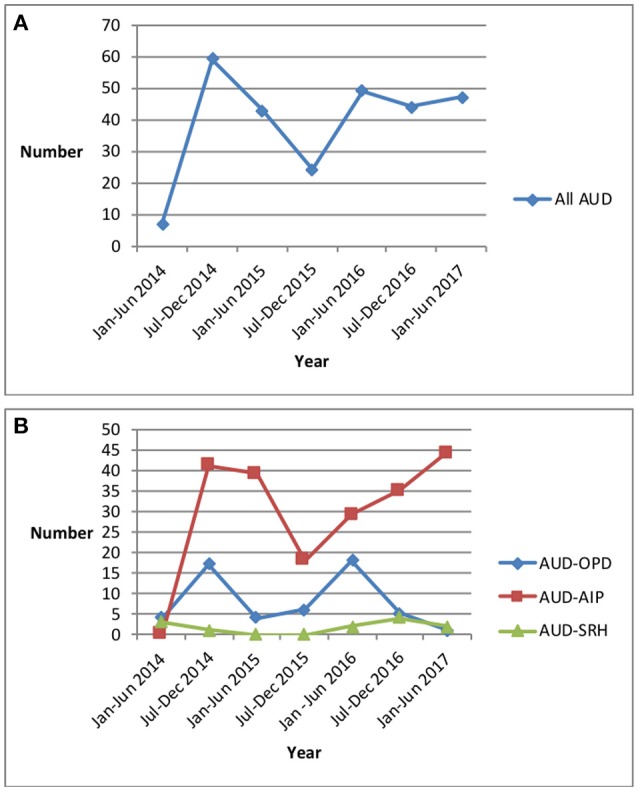
Trends in numbers of patients diagnosed in 6-month periods with Alcohol Use Disorders in Bwindi Community Hospital, Uganda, between January 2014 and June 2017. **(A)** All patients at BCH with Alcohol Use Disorder. **(B)** Patients with Alcohol Use Disorder in the three different departments at BCH. AUD, Alcohol Use Disorder; AUD-OPD, Alcohol Use Disorder in Out-patient Department; AUD-AIP, Alcohol Use Disorder in Adult In-Patient Department; AUD-SRH, Alcohol Use Disorder in Sexual & Reproductive Health Department; BCH, Bwindi Community Hospital.

Trends in the numbers with AUD in the 6-month periods between 2014 and 2017 are shown in Figure 1. There was some variation in the total counts with the numbers with AUD between January 2016 and July 2017 being fairly consistent at between 44 and 49 per 6-month periods (Figure 1A). For the three different departments, the main findings were a decrease in AUD in the out-patient and an increase in AUD in the adult in-patient departments between January 2016 and June 2017 (Figure 1B).

Overall, the numbers and proportions of those with different AUD scores were as follows: patients with score 8–15 (increased risk): *n* = 147 [54%]; patients with score 16–19 (higher risk): *n* = 46 [17%]; patients with score 20 or above (possible dependence): *n* = 79 [29%]. In the out-patient department, there were more patients with possible dependence [26 of 55, 47%]. In the other two departments there were more patients with increased risk [116 of 206, 56% in adult in-patients, and 11 of 12, 92% in sexual & reproductive health].

The majority of those in the out-patient and adult in-patient departments were male with the most common age range being 36–60 years (Table 2). Overall, the most common clinical diagnostic category of those with AUD was non-alcohol related medical disease. Seeking help for alcoholism was the second most common category in those attending out-patients while surgical non-alcohol related disease was the second most common category in adult in-patients.

**Table 2:** Demographic and clinical diagnostic categories in patients diagnosed with Alcohol Use Disorders in three different departments of Bwindi Community Hospital, Uganda, between January 2014 and June 2017.

**Characteristics**	**Out-patient department**		**Adult in-patient department**		**Sexual and reproductive health department**		**Total**	
	***n***	**(%)^a^**	***n***	**(%)^a^**	***n***	**(%)^a^**	***n***	**(%)^a^**
Total	55	(100)	206	(100)	12	(100)	273	(100)
**AGE GROUP—YEARS**								
19–35	19	(35)	71	(34)	12	(100)	102	(37)
36–60	24	(44)	106	(51)	0	(0)	130	(48)
60 and above	12	(21)	29	(15)	0	(0)	41	(15)
**GENDER**								
Male	54	(98)	176	(85)	0	(0)	230	(84)
Female	1	(2)	30	(15)	12	(100)	43	(16)
**CLINICAL DIAGNOSTIC CATEGORY**								
• Medical—alcohol related^b^	2	(4)	32	(16)	0	(0)	34	(12)
• Medical—not alcohol related	34	(62)	95	(46)	2	(17)	131	(48)
• Surgical—alcohol related^c^	0	(0)	14	(7)	0	(0)	14	(5)
• Surgical—not alcohol related	1	(2)	39	(19)	0	(0)	40	(15)
• Pregnancy and/or labor	0	(0)	0	(0)	3	(25)	3	(1)
• Seeking help for alcoholism	15	(27)	4	(2)	0	(0)	19	(7)
• Not recorded	3	(5)	22	(10)	7	(58)	32	(12)

a *Column percentages*.

b *Includes cirrhosis of the liver (clinically diagnosed), alcohol-induced hypoglycemia, alcohol-induced psychosis, or acute intoxication*.

c *Includes road traffic accidents, trauma, or burns while under the influence of alcohol*.

The medical and supportive interventions which were given to patients with AUD in order of overall frequency are shown in Table 3. Brief guidance and counselling followed by social support from community members were the two most common interventions, followed by referral to mental health services, detoxification and referral to AA. There were some variations in the frequency of these services by different departments, with detoxification occurring in nearly one in five adult in-patients.

**Table 3:** Medical and supportive interventions for patients diagnosed with Alcohol Use Disorders in three different departments of Bwindi Community Hospital, Uganda, between January 2014 and June 2017.

**Medical and supportive interventions**	**Out-patient department**		**Adult in-patient department**		**Sexual and reproductive health department**		**Total**	
	***n***	**(%)^a^**	***n***	**(%)^a^**	***n***	**(%)^a^**	***n***	**(%)^a^**
Total with alcohol-use disorder	55	(100)	206	(100)	12	(100)	273	(100)
Brief guidance and counseling	41	(75)	125	(61)	2	(17)	168	(62)
Social support from community members	32	(58)	57	(3)	1	(8)	90	(33)
Referral to Mental Health Team	42	(76)	32	(16)	1	(8)	75	(27)
Detoxification^b^	22	(40)	37	(18)	1	(8)	60	(22)
Referral to alcoholics anonymous	20	(36)	20	(8)	1	(8)	41	(15)

a *Column percentages—these do not add up to 100% because patients could receive more than one intervention*.

b *Vitamin B1 supplementation (oral and/or intramuscular), benzodiazepines, anti-psychotic medication if there are hallucinations or other forms of psychosis*.

The numbers and proportion of patients with both AUD and a clinical diagnostic category who received no medical or supportive interventions are shown in Table 4. Altogether nearly 15% of such patients received no interventions for AUD. Using those diagnosed with medical alcohol related disease as the reference standard, patients with surgical alcohol-related disease and women with pregnancy-related conditions were significantly more likely to receive no AUD intervention (*P* < 0.03 and *P* < 0.02, respectively).

**Table 4:** Number and proportion of patients with Alcohol Use Disorders who received no medical or supportive interventions in relation to their clinical diagnostic categories in Bwindi Community Hospital, Uganda, between January 2014 and June 2017.

**Clinical diagnostic category**	**All patients with AUD**	**Patients receiving no medical or supportive intervention**		**OR (95% CI)**	***P*-value**
	***n***	***n***	**(%)^a^**		
Total with alcohol-use disorder^b^	241	36	(14.9)	NA	NA
Medical—alcohol related	34	2	(5.9)	ref	ref
Medical not alcohol related	131	20	(15.3)	2.9 (0.6–13.0)	0.15
Surgical—alcohol related	14	4	(28.6)	6.4 (1.0–40.3)	0.03
Surgical—not alcohol related	40	8	(20.0)	4.0 (0.8–20.3)	0.08
Pregnancy and/or labor	3	2	(66.7)	32.0 (2.0–522)	0.02
Seeking help for alcoholism	19	0	(0)	–	–

a *Row percentages*.

b *Includes only those patients with a known clinical diagnostic category*.

*AUD, alcohol use disorders; OR, odds ratio; CI, confidence intervals; NA, not applicable*.

## Discussion

This is the first study from BCH to assess the relatively new programme designed to screen, diagnose, and manage persons with AUD. There were three main interesting findings.

First, about 10% of all those who attended or were admitted to one of the three departments were screened for AUD with <5% of those screened being diagnosed with the condition. In terms of screening and diagnostic yield, the results were best from the adult in-patient department with numbers of AUD increasing in the last 18 months. Possible reasons for the superior performance of the adult in-patient department were the lower numbers of admissions compared with the other two departments and therefore more potential time to screen patients and in the last few months of the study the introduction of mandatory screening for AUD before hospital discharge. Conversely, the lower proportion of patients screened in the out-patient department was probably related to the high numbers of attendances with limited time and opportunity for staff to undertake AUD screening. The majority of those diagnosed with AUD were male with half having a medical non-alcohol related clinical diagnosis. These findings are in line with recent national publications about males being more affected than females and more likely to suffer from AUD (1, 14).

Second, brief guidance, counselling and community-based social support were the most common medical and supportive interventions given. About a quarter of patients with AUD were referred to the mental health team, with smaller proportions receiving detoxification or being referred to AA. Counselling and social support are the easiest interventions to offer in a low-resource setting such as Bwindi which probably explains why they were commonly offered. Unfortunately no further details were possible from the routinely collected data set about why patients were detoxified, referred to the mental health team or AA.

Third, about 15% of patients with AUD received no intervention at all, and this was higher in patients with surgical-alcohol related diseases and pregnant women. While it is surprising that those with surgical-alcohol related diseases had no intervention, it is possible that the surgical problems received much higher priority than the AUD interventions and the latter were either not offered or were offered but not documented. The same considerations apply to pregnant women delivering babies.

The key strength of this study was that screening, diagnosis and management of AUD were embedded into the health services of three departments of BCH, and the results therefore reflect routine practice. Two other strengths were that we used the WHO-based AUDIT tool to diagnose AUD (10, 20) and we conducted and reported the study in line with the Strengthening the Reporting of Observational Studies in Epidemiology (STROBE) (25). There were a number of limitations related to the observational design and use of secondary data which limited our information about variations in administration of procedures and the fidelity of clinical and nursing staff to screening, diagnosis and management procedures across the hospital departments. There was lack of specific detail around socio-demographic and clinical characteristics of patients diagnosed with AUD. Going forward it would be important to try and collect and/or analyze more details on other clinical and environmental risk factors that are associated with AUD (26, 27). Finally, we do not have information about what the clinical or nursing staff on the ground thought about AUD screening or their insights about the low uptake in the out-patient department. This is an area for further research.

Two areas require further comment. First, detoxification with specific medications was given to one fifth of patients. The major reason for detoxification is to help patients cope with the symptoms and signs related to alcohol withdrawal. There are several drugs available for the treatment of AUD that include medications to reduce or stop severe alcohol withdrawal symptoms (28) and BCH has a small number of these medications for current use. There are also specific longer-term medications that aim to reduce alcohol craving and support abstinence (28), but a recent Cochrane review concluded that there was no sound evidence that these are useful (29). This supports the BCH philosophy of offering brief guidance, counselling and community social support to try and effect behavioral change rather than provide long-term medical treatment. Any patient, however, with an associated mental health disorder is referred to the mental health team where specific medication for a diagnosed psychiatric condition may be given.

Second, the formation of AA groups that visit the hospital or provide support in the community started following the screening, diagnosis and management program for AUD. Alcoholics Anonymous is an international mutual aid fellowship, founded in 1935, with the primary purpose of enabling those with alcohol use problems to stay sober as a result of adhering to the “Twelve Steps” (30). The effectiveness of AA is controversial, with some scientific studies citing benefit with short- and long-term decreases in alcohol consumption (31, 32) while others have demonstrated little to no effect (33). The current study documented the number of persons with AUD referred to AA groups, but we have no further information about the quality or effectiveness of this intervention or about the acceptability of this form of support in the community. Further in depth, qualitative research is needed in this area.

There are three important implications arising from this study. First, it is possible to improve the screening process. The adult in-patient department is where numbers being screened are above 50% and where the greatest numbers of patients are being diagnosed with AUD. In April 2017, it became mandatory in this department to screen all patients for AUD before discharge with the hospital electronic data system refusing to allow the entry of “hospital discharge” until the screening has been carried out. A further audit of this new approach is required and planned from mid-2018 onwards. In the out-patient department, there is currently no systematic screening and this is reflected in the low numbers of patients screened for AUD. This could improve with a more targeted systematic screening approach either instigated by health care workers or requested by the patients themselves as a result of more public awareness of the problem.

Second, it is essential that medical and supportive interventions are given to all those with AUD, and it is unacceptable that 15% of patients in our study received no help. Going forward, the hospital could introduce regular audits every quarter of the number and proportions of those with AUD receiving some form of help and a target set that 100% of patients should receive one or more medical/supportive interventions. More detailed qualitative research is also needed about the type and effectiveness of mental health treatment provided and about the functioning of the hospital-focused and community-based AA groups, especially from the patients' perspective.

Third, we need to step up public awareness in the community's 101 villages about the harmful use of alcohol. This is already being done through the community mental health program and community health volunteers. However, this could be strengthened by better and more regular engagement with village headmen, other traditional authorities, traditional healers and church organizations and we need more use of the radio to disseminate public health messages about the harmful effects of alcohol and where to seek help if needed. Furthermore, we need to also share the concept behind this intervention with other stakeholders, especially policy makers both at the ministry and district level for possible replication at other hospitals. The staff of all the hospitals involved could then have regular meetings, share their experiences and forge a way forward for the continuity of the intervention.

## Conclusion

This study in BCH, rural Uganda, assessed a program of screening, diagnosis and management of patients with AUD between January 2014 and June 2017. About 10% of all those who attended or were admitted to one of three departments of the hospital were screened with <5% of those screened being diagnosed with AUD. The best results were from the adult in-patient department in terms of screening and diagnostic yield. While a variety of different medical and supportive interventions were given to those with AUD, mainly focused on brief guidance, counselling and community social support, 15% of patients received no intervention at all, and this was higher in surgical patients and pregnant women. Policy implications to improve program performance include better screening processes, ensuring that 100% of those diagnosed with AUD receive a medical or supportive intervention and raising public awareness.

## Author contributions

YK conceived the study; YK, AD, AH, BM, AS, and EW contributed to designing the study protocol; YK and AD collected the data; YK, AD, and AH analyzed and interpreted the data; YK and AH wrote the first draft of the manuscript; YK, AD, AH, BM, AS, and EW critically revised the manuscript for intellectual content; YK, AD, AH, BM, AS, and EW read and approved the final manuscript; YK is guarantor of the paper.

## Data availability statement

Data sets are available on request. The raw data that support the results and conclusions of the study can be made available, without undue reservation, to any qualified researcher.

### Conflict of interest statement

The authors declare that the research was conducted in the absence of any commercial or financial relationships that could be construed as a potential conflict of interest.
